# The Neurobiological Basis of Love: A Meta-Analysis of Human Functional Neuroimaging Studies of Maternal and Passionate Love

**DOI:** 10.3390/brainsci12070830

**Published:** 2022-06-26

**Authors:** Hsuan-Chu Shih, Mu-En Kuo, Changwei W. Wu, Yi-Ping Chao, Hsu-Wen Huang, Chih-Mao Huang

**Affiliations:** 1 Department of Biological Science and Technology, National Yang Ming Chiao Tung University, Hsinchu 300093, Taiwan; shihhc12@gmail.com (H.-C.S.); kuo.muen@gmail.com (M.-E.K.); 2 Center for Intelligent Drug Systems and Smart Bio-Devices (IDS2B), National Yang Ming Chiao Tung University, Hsinchu 300093, Taiwan; 3 Institute of Biomedical Engineering, National Yang Ming Chiao Tung University, Hsinchu 300093, Taiwan; 4 Graduate Institute of Mind, Brain, and Consciousness, Taipei Medical University, Taipei 106052, Taiwan; restingfmri@gmail.com; 5 Department of Computer Science and Information Engineering, Chang Gung University, Taoyuan 33302, Taiwan; catpin@gmail.com; 6 Department of Linguistics and Translation, City University of Hong Kong, Hong Kong, China; hwhuang@cityu.edu.hk; 7 Institute of Brain Science, National Yang Ming Chiao Tung University, Taipei 112304, Taiwan

**Keywords:** human functional neuroimaging, meta-analysis, maternal love, passionate love, ventral tegmental area (VTA), activation likelihood estimation (ALE)

## Abstract

Maternal and passionate love are both crucial for reproduction and involve attachment behaviors with high rewards. Neurobiological studies of attachment in animal and human neuroimaging studies have suggested that the coordination of oxytocinergic and vasopressinergic pathways, coupled with the dopaminergic reward system, contribute to the formation and maintenance of maternal and passionate love. In the present study, we carried out a quantitative meta-analysis of human neuroimaging to identify common and dissociable neural substrates associated with maternal and passionate love, using the activation likelihood estimation (ALE) approach. The ALE results showed significant activation of the brain regions in the left ventral tegmental area (VTA), right thalamus, left substantia nigra, and the left putamen for maternal love, but in the bilateral VTA for passionate love. The meta-analytic neuroimaging evidence suggests the greater involvement of cognitive–affective regulation in maternal attachment and the greater desire to combine liking and wanting in romantic love behaviors. The conjunction analysis highlights the functional convergence of the VTA across the two types of human love, indicating a shared neurobiological mechanism of maternal and passionate love with evolutionary roots. Our findings suggest that the processing of both maternal and passionate love involve the affective and motivational regulation associated with dopaminergic systems; our neuroimaging evidence supports the notion that maternal and passionate love share a common evolutionary origin and neurobiological basis in the human brain.

## 1. Introduction

For many decades, social and biological scientists have been trying to identify the nature of human love, which is of critical evolutionary importance for reproduction. In 1958, psychologist John Bowlby proposed his attachment theory as an underlying principle of the nature of love. He posited that primate infants develop an emotional attachment with their primary caretakers to protect them from danger and that human infant behavior is also regulated by this emotional strategy; it helps them to build relationships with friends, partners, and later, their own infants [1].

To further understand how exactly love works as a neurobiological mechanism, several animal experiments have been undertaken. Previous animal studies have suggested that oxytocin, vasopressin, and dopamine play important roles in mammal attachment [2,3]. Oxytocin and vasopressin are neurotransmitters whose composition differs by only two amino acids and whose neurobiological mechanisms often manifest along the same pathway. Oxytocin is produced in the hypothalamus and released from the posterior pituitary to the amygdala, brain stem, or autonomic nervous system, producing emotions such as fear, anxiety, and/or aggression [4,5]. However, more complex consequences of oxytocin and vasopressin have been uncovered in this interaction, as exogenous oxytocin was found to increase aggressive behaviors in hamsters, which could be attenuated by blocking their vasopressin receptors. Ultimately, however, the only neurobiological response needed to achieve social reward was oxytocin, which acts on oxytocin receptors in the ventral tegmental area (VTA) [6,7,8].

The VTA is in the midbrain, the front portion of the brain stem. Unlike other highly developed regions, the function of the VTA is likely primitive, such as motor control, locomotion, and addiction [9,10]. However, studies of the midbrain dopamine system have shown that VTA dopaminergic neurons also affect motivation and reward-related behaviors [11,12]. Based on this evidence of the midbrain dopamine system’s diversity, we hypothesize that, from an evolutionary perspective, the VTA may be involved in complicated interactive processing between goal-directed cognitive function and reward-related social behaviors in humans; in this case, maternal and/or passionate love.

In the regulation of their emotional response to threats through oxytocin and vasopressin pathways, mammals achieve safety by developing attachments with others [13,14]. An indispensable part of developing attachment is motivation, a driving force that utilizes the dopaminergic system. A study of rats found that, through the action of dopamine in the nucleus accumbens (NA), the NA–ventral pallidum (NA–VP) circuits are stimulated to activate motivation in the infant and care partner. Moreover, oxytocin is involved in this dopaminergic pathway via oxytocin signaling [15,16] that strengthens synaptic plasticity when the infant’s and partner’s NA–VP circuits are stimulated. Evidence also suggests that the amount of oxytocin receptors in the NA depends on maternal care during this sensitive period; losing the partner suppresses oxytocin signaling in the NA [17,18]. Such coordination of dopamine and oxytocin in the NA associated with a valued social affiliation was also reported in rhesus macaques [19]. These convergent findings in molecular neuroscience suggest that, from an evolutionary perspective, maternal behavior and pair-bonding may share similar neurobiological mechanisms of human attachment.

Although the behaviors of social bonding stem from the quality of the attachment a mother builds with her infant during the early sensitive period [13], human bonds and attachment are plastic and complicated enough for a later secure attachment to modify an early insecure one [20]. To further investigate the mystery of human attachment, some studies used a human neuroimaging approach. In one early study, Bartels and Zeki [21] used functional magnetic resonance imaging (fMRI) techniques to measure differences in the brain activity of participants as they viewed significant romantic partners versus friends. They found passionate love to be more complicated than basic emotions, involving the highly motivating reward of evoking an overwhelming response in a partner [22]. Later, they measured the neural response of mothers to their own infants using fMRI to assess the similarity of neural mechanisms between these two highly rewarding experiences involving firm bonds [22]. Interestingly, although the activated regions did not overlap much, they found that both maternal and passionate love involve an activated reward circuitry consisting of the putamen, globus pallidus, caudate nucleus, and VTA, which contain dopamine and oxytocin receptors. In addition, both kinds of love deactivated the amygdala and mesial prefrontal cortex, which regulate negative emotions and social judgement. These results suggest that human attachment behaviors may share a similar neurobiological mechanism and evolutionary origin to animal attachment, but with a more advanced involvement of cognition.

After maternal and passionate love were preliminarily identified using this functional neuroimaging approach and the attachment-mediated neurotransmitters defined, a meta-analysis was conducted of maternal, passionate, and unconditional love (a total of six fMRI studies) to compare different patterns of multivariate human attachment [23]. The study found that passionate love recruited regions of the brain that mediate motivation, emotion, social cognition, and self-representation, such as the VTA, caudate nucleus, anterior cingulate gyrus, and middle frontal gyrus; maternal love activated similar patterns in motivation, emotion, and cognition, but specifically activated the periaqueductal gray matter and other regions dealing with high-order cognition and emotional processing; interestingly, unconditional love—the definition of which was not confirmed—recruited neural circuity similar to that of maternal love. As a final point, although different types of love were found to have unique cerebral networks, all of them activated goal-directed motivation, dominated by a reward system and corresponding regions full of dopamine and oxytocin receptors.

The realm of human attachment has been predominantly defined as the interpersonal relationship [21,22,23]. A recent study extends the concept of attachment to the relationship between a person and an object and conducted a meta-analysis of love to investigate neural substrates of brand love which has been shown to reveal strong ties between consumers and brands (i.e., “brand attachment”) and compared it with those of maternal and romantic love [24]. The study, when the loose criterion was applied (*p* < 0.001 uncorrected), reported that the brain regions of putamen and insula were commonly recruited for brand love, maternal love, and passionate love. However, brand love appears to show different dispositions from the other two types of interpersonal love, with the brain activation of dorsal striatum for brand love whereas distributed cortical areas and globus pallidus for maternal love and NA and VTA for passionate love [24]. These results highlight the similarity and discrepancy between brand love and interpersonal love, however, the common and dissociable neutral substrates associated with maternal and passionate love from the neurobiological and evolutionary perspectives remain unclear.

In our study, a quantitative meta-analysis of fMRI studies was carried out in relation to maternal and passionate love to identify the stable regions of brain activity engaged during each type of human love and across these two types. We also aimed to identify differences in reliable brain activity between maternal and passionate love using the activation likelihood estimation (ALE) method. We hypothesized that maternal love and passionate love share a similar distribution of brain networks related to affective, motivational, and cognitive processing, centering on the VTA. Moreover, maternal brain activity may involve more regions abundant in oxytocin and vasopressin receptors, which are crucial for maternal–infant bonds, than passionate love.

## 2. Methods

### 2.1. Systematic Review and Selection of Studies

**Figure 1:**
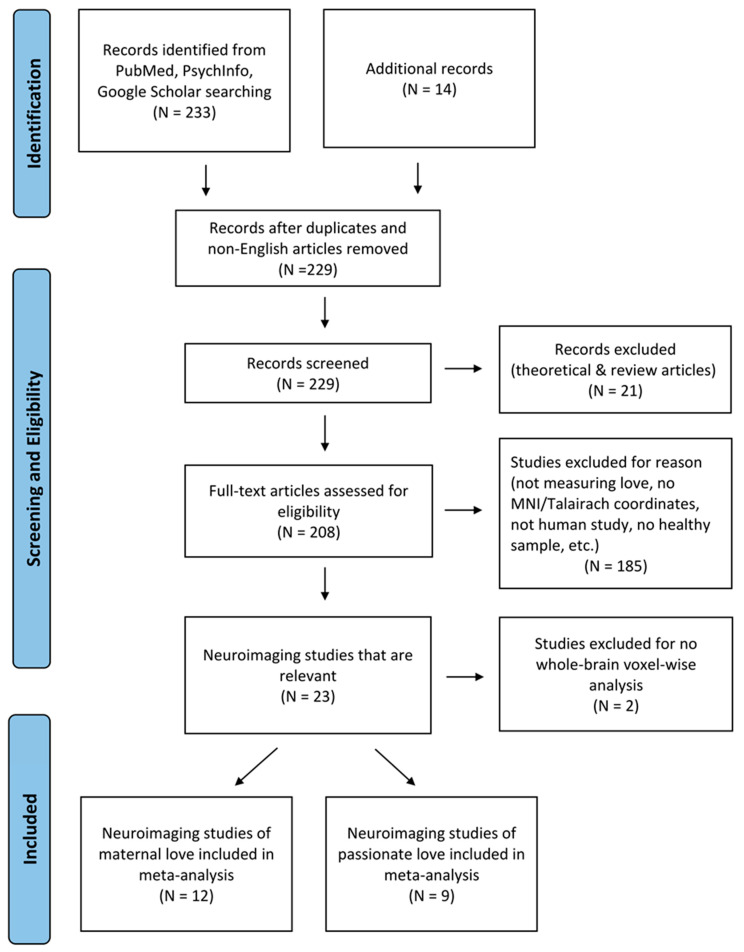
The PRISMA flowchart of literature search and study selection for meta-analysis of human love.

Human neuroimaging studies of maternal and passionate love were selected using a systematic search process. Peer-reviewed articles published in English between January 1997 and July 2021 were searched from the National Center for Biotechnology Information (NCBI) PubMed database (i.e., Medline), PsychInfo, and Google Scholar, using the following keyword combinations: (“romantic love” or “passionate love” or “maternal” or “love”) and (“fMRI” or “functional magnetic resonance imaging” or “neuroimaging” or “PET” or “positron emission tomography”). In addition, we searched the peer-reviewed articles listed in relevant papers through the “similar article” function in the PubMed database and the reference lists in each relevant paper. As a result, 102 unique papers were found. Two of the authors (H.C. Shih and C.M. Huang) confirmed the inclusions of the identified studies, and the search of literature and selection of studies process are visualized in Figure 1.

We selected studies that satisfied the following criteria: (1) neuroimaging studies reporting complete Montreal Neurological Institute (MNI) and/or Talairach coordinate systems and (2) studies identifying participants as “loving” partners, mothers, or infants. For example, for maternal love, we excluded peer-reviewed articles about mothers with postpartum depression or who had been in deep conflict with their husbands; for passionate love, we included only peer-reviewed articles that provided participants’ ratings on the Passionate Love Scale [25]. Moreover, we excluded theoretical papers and reviews. Twelve maternal love fMRI studies and nine passionate love fMRI studies were ultimately included (Table 1). For studies containing multiple independent experiments, the peak activation foci from each experiment were identified, resulting in 29 experiments for maternal love and 13 experiments for passionate love. The 29 experiments for maternal love differed in the valence of emotional stimuli (e.g., positive, neutral, and negative) and across different domains of stimuli (i.e., visual and auditory). The 13 experiments for passionate love involved long-term intense romantic love, with participants having been married more than 10 years but still having a good rating on the Passionate Love Scale [26]. Moreover, the experiments for passionate love varied across sexual orientation (i.e., heterosexual and homosexual passionate love) and cultural groups (e.g., Chinese and Westerners).

### 2.2. Meta-Analysis: ALE

The Activation Likelihood Estimation (ALE) method provides a voxel-based quantitative meta-analytic approach to functional neuroimaging data. This meta-analysis of human functional neuroimaging data was conducted using an algorithm from the BrainMap GingerALE software, version v2.3.6 [45,46,47], which was developed to improve the ALE method [48]. GingerALE computes the statistically significant concordance of brain activity across several independent experiments and between two sets of independent experiments. In GingerALE analysis, all activation foci reported as three-dimensional coordinates in stereotactic space were modeled with a three-dimensional Gaussian probability distribution. The maximum probabilities of activation were then calculated to create modeled activation maps for each independent experiment and followed by computing the union of all modeled activation map via voxel-by-voxel approach while sample size in each independent experiment were considered. The whole-brain ALE maps were created within and across independent experiments by comparing null hypothesis maps representing the noise distribution. The reliability of whole-brain ALE maps was determined by applying a permutation procedure to recognize the differences between true convergence of activation foci and random clustering [45,46,47].

Five separate GingerALE analyses were conducted to create significant whole-brain ALE maps and corresponding activation clusters: (1) significant patterns of brain activation associated with maternal love; (2) significant patterns of brain activation associated with passionate love; (3) significant patterns of brain activation shared by maternal and passionate love (i.e., maternal love ∩ passionate love); and (4) differences in brain activation patterns between maternal and passionate love across all studies (i.e., maternal love vs. passionate love). The studies that contributed these foci are presented in Table 1. The foci were modeled using a full-width half maximum (FWHM) of 6 mm^3^ to calculate 3D Gaussian probability distributions. To create the ALE maps and identify peak activation locations, the threshold of significance was set to a family-wise error rate (FWE) of *p* < 0.05 with volume > 200 mm^3^.

## 3. Results

A total of 29 experiments with 528 foci across 12 neuroimaging studies of maternal love and 13 experiments with 169 foci across 9 neuroimaging studies of passionate love were identified (Table 1). Table 1A,B contain details of the included studies, including numbers of participants, experimental stimuli, contrasts, the numbers of foci, for maternal and passionate love, respectively

### 3.1. ALE Results for Maternal Love

**Figure 2:**
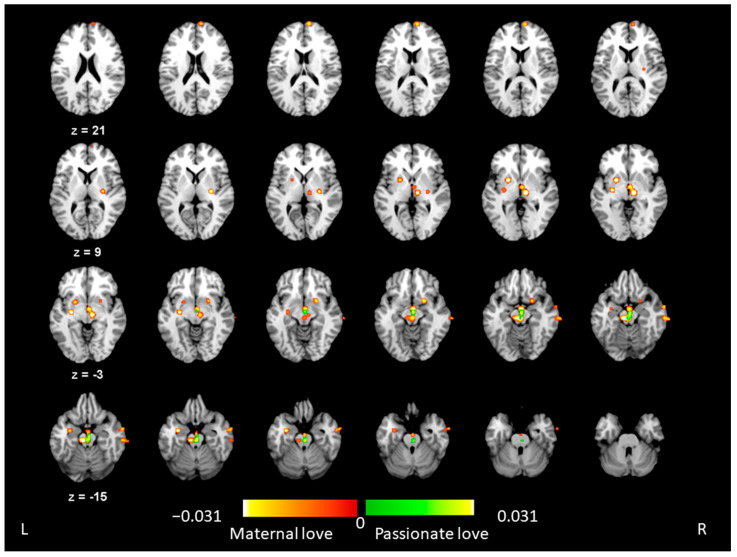
Activation likelihood clusters (FDR pN < 0.01 level) show meta-analytic activations in maternal love (red) or passionate love (green). In maternal love, the activated clusters were centered at the left ventral tegmental area, right thalamus, left substantia nigra, bilateral putamen, right medial prefrontal gyrus, right superior temporal gyrus, right middle temporal gyrus, and left amygdala. A cluster was activated in the meta-analysis of passionate love, centered at the left ventral tegmental area and extending to the right ventral tegmental area.

Table 2 lists the coordinates of the maxima for the activated brain regions associated with maternal love. Five clusters were significant in the meta-analytic results of maternal love across studies at the FWE *p* < 0.05 level. The centers of each cluster were the left VTA, right thalamus, left substantia nigra, and left putamen. When the threshold of significance was set to a false discovery rate (FDR) of pN < 0.01, five more clusters were activated [49]: the right putamen, right medial prefrontal gyrus, right superior temporal gyrus, right middle temporal gyrus, and left amygdala (Table 2). The ALE maps (FDR pN < 0.01) presenting all the activated brain regions associated with maternal love are shown in Figure 2.

### 3.2. ALE Results for Passionate Love

Table 3 lists the coordinates of the maxima for the activated brain regions associated with passionate love. One cluster was significantly activated in meta-analysis across all the relevant passionate love studies (FWE *p* < 0.05, volume > 200 mm^3^), centered at the left VTA and extending to the right VTA. Figure 2 also shows the ALE maps (FDR pN < 0.01) for all the activated brain regions associated with passionate love.

### 3.3. ALE Conjunction Results across Maternal and Passionate Love

**Figure 3:**
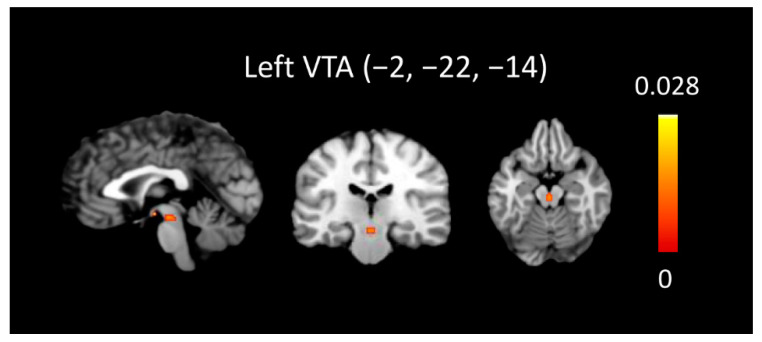
ALE map of conjunction analysis on maternal love with passionate love (FDR pN < 0.01).

To identify the common neural substrates shared by maternal and passionate love (i.e., maternal love ∩ passionate love), a conjunction analysis of activation likelihood methods was performed across the two types of human love by computing the intersection between the meta-analyses on maternal and passionate love. There was no significant result at the level of FWE *p* < 0.05. The conjunction analysis returned one cluster centered at the left VTA (x = −2, y = −22, z = −14) when the threshold was set to the level of FDR pN < 0.01. Figure 3 shows the ALE map of conjunction analysis on maternal love with passionate love.

### 3.4. ALE Results Indicating Differences between Maternal and Passionate Love

To reveal the differences in neural signatures between maternal and passionate love, a contrast-analysis of activation likelihood methods was performed between the two types of human love by contrasting studies on maternal love with those about passionate love. There was no significant result at the level of FWE *p* < 0.05 for both contrasts (i.e., maternal > passionate love; passionate > maternal love). When the threshold was set to the level of FDR pN < 0.01, however, we found one cluster located at the left putamen that showed greater activation for maternal love than for passionate love, but no significant cluster was observed for passionate love than for maternal love. Table 4 lists the coordinates of the maxima in the activated region when contrasting the two types of love.

## 4. Discussion

This quantitative meta-analysis of functional neuroimaging studies on maternal and passionate love in humans love yields three main findings. First, significantly activated brain regions associated with maternal love were found in the left VTA, right thalamus, left substantia nigra, and left putamen. The brain regions associated with passionate love were reported in bilateral VTAs, indicating the highly rewarding nature of human romantic relationships. Second, both maternal and passionate love appeared to be associated with functional activations in the left VTA, indicating the engagement of evolutionarily stable brain mechanisms across the two types of human love. Finally, a direct comparison between maternal and passionate love showed a stronger putamen activation for maternal love than for passionate love, suggesting that putamen may play a critical role to connect to the subcortical and cortical regions for a greater degree of cognitive–emotional regulation of attachment behavior in maternal attachment.

Contrary to previous results [22,24], we found brain activation of the right medial prefrontal gyrus, right superior/middle/inferior temporal gyrus, and left amygdala when ALE methods were applied across several maternal love studies. These regions have been associated with social and emotional judgement, unless their functions were damaged by lesions [50]. The mesial prefrontal cortex has been associated with the temporal cortex and contributes to the “theory of mind” involved in information processing of others’ emotions [51,52]. Moreover, as the amygdala has been identified as playing a critical role in processing emotion, its reliable activation may coordinate and interact with the “theory of mind” network to establish and maintain maternal attachment through appropriate emotional expression and responses between infants and mothers. Such cognitive–affective control functions highlight the evolutionary need for “maternal sensitivity,” a crucial indicator for secure or insecure attachment [53]. Therefore, our findings advance our understanding of human love beyond the push–pull mechanism proposed by Bartels and Zeki [22] and our meta-analytic results suggest that human love may involve more widely distributed regions of the brain in the cognitive–affective control processes.

Given that the meta-analytic results showed significantly activated brain regions in the VTA, thalamus, and substantia nigra, these findings point to the importance of reward signals in maternal love. The dopaminergic reward system has been identified as being rich in dopaminergic neurons located in the VTA, substantia nigra, striatum, NA (basal ganglia), and ventral prefrontal cortex. It has been shown to respond across various domains of rewarding experiences, such as money, cocaine, and sex [54,55]. It is likely that the reward system is predominant in human love as the feelings involved in obsessive and euphoric behavior recruit the basal ganglia [56]. More importantly, through modifications of synaptic plasticity, the dopaminergic reward system also plays an important role in learning and memory, resulting in stronger memory consolidation that contributes to the attachment experience [57]. Evidence from primate studies has shown that certain dopaminergic neurons in the striatum encode a reward signal from interactions between people and find rewards specifically in interactive behavioral patterns [19]. In addition to the dopaminergic pathway, the activated regions of the hypothalamus, VTA, and caudate nucleus reported in this meta-analysis also suggest the involvement of oxytocinergic and vasopressinergic pathways in attachment behaviors and social interactions, which may act as modulators of human love [58]. Previous animal experiments have shown that oxytocinergic lesions or knockout techniques directly cause the loss of appropriate maternal behaviors in rats [59]. Our study, therefore, provides supportive meta-analytic neuroimaging evidence that associates material love in human with the neural reward system in coordination with the dopaminergic and oxytocinergic pathways, creating unique patterns that are different from basic emotions.

Furthermore, our meta-analysis of passionate love showed bilateral VTA activation. It has been suggested that there are hemispheric differences in the function of the VTA, with the right VTA associated with the behavior of “wanting” and the left VTA associated more with “liking” [38,43]. Our quantitative meta-analysis reported a left-hemispheric VTA activation for maternal love and bilateral VTA activation for passionate love. This finding may suggest a greater desire to combine liking and wanting in romantic love behaviors. An fMRI study focusing on long-term intense romantic love [26] reported that the right VTA was correlated with the Inclusion of the Other in the Self (IOS) scale, especially for romantic partners and friendships, highlighting closeness in attachment and pair-binding.

The conjunction analysis on two types of human love, maternal and passionate love significantly converged in the left VTA. This finding suggests a neurobiological mechanism of maternal and passionate love with common evolutionary roots. Previous studies have shown neurobiological evidence in the neural circuit and at a molecular level, suggesting that specific neurotransmitters in the mesocorticolimbic system, including the VTA, support the establishment and maintenance of social bonds. In maternal love, the behavior of recognizing one’s infant also imparts warmth, comfort, food, and protection. Shahrokh’s (2010) study of rat models clearly shows the direct effect of oxytocin at the level of the VTA in the regulation of NA dopamine levels, which drives mothers to increase pup licking and grooming [60]. A comparison of frequently licking/grooming mothers with infrequently licking/grooming mothers shows greater increases in the dopamine signal in the NA during bouts of pup licking/grooming. This difference is abolished upon the direct infusion of an oxytocin receptor antagonist into the VTA [60,61]. Passionate love involves special feelings for a specific other, related to pair bonding behavior in monogamous species, including humans. In studies of animal models, our understanding of the neural mechanism underlying pair bonding mostly comes from prairie voles. Liu’s (2003) study has shown that the activation of oxytocin and dopamine D2-type receptors in the NA, a strong projection target of the VTA, is essential for pair bond formation [62]. Despite the well-known functions of the VTA in desire, reward prediction error, and/or decision-making, our meta-analysis further suggests that neural activity in the VTA is essential for both maternal and passionate love.

In conclusion, this study suggests that the brain networks associated with affective and motivational processing for perceiving maternal and passionate love are similar but distributed differently. Neuroimaging evidence confirms that these two types of love share a common evolutionary origin and neurobiological mechanism. Although our meta-analysis included neuroimaging studies with diverse experimental designs and participant demographics that may have undermined its potential effects, the ALE approach we used to review the convergence of brain mechanisms of human love took advantage of statistical power to conclude its domain- and culture-invariant effects and demonstrated the characteristics of maternal and passionate love. By including every available functional neuroimaging study on human love, we were able to present a common basis for these two types of love. Future directions should include a clearer definition of human love, various types of human attachment, and a neuroscientific approach to love.

## Figures and Tables

**Table 1:** (**A**): Details of included studies on maternal love. (**B**): Details of included studies of passionate love.

**(A)**								
**First Author**	**Year**	**Categories of Love**	**Numbers of Participants**	**Mean Age of Participants’ Own Child**	**Mean Age of Participants**	**Experimental Stimuli**	**Contrasts**	**Numbers of Foci**
Lorberbaum [27]	2002	Maternal	10	6.18 weeks	30.55	Cry sound and white noise (auditory)	Infant cry > Rest	80
Bartles [22]	2004	Maternal	20	24.4 months	34	Pictures (visual)	Own child > Acquainted child	28
Nitschke [28]	2004	Maternal	6	3–5 months	No report	Pictures of faces (visual)	Own infant > Unfamiliar infant	6
Leibenluft [29]	2004	Maternal	7	5–12 years	32.9	Pictures of faces (visual)	Own child > Familiar child	36
Ranote [30]	2004	Maternal	10	25.6 months	26	Video Clip (visual-auditory)	Own infant > Unknown infant	3
Noriuchi [31]	2008	Maternal	13	16.5 months	31.1	Video Clip (visual-auditory)	Own infant > Other infant	81
Lenzi [32]	2009	Maternal	16	9.5 months	33.7	Pictures of faces (visual)	Own child > Acquainted child	7
Strathearn [33]	2008	Maternal	28	6.7 months	30.2	Pictures of faces (visual)	Own infant > Unknown infant	67
Strathearn [34]	2009	Maternal	30	7 months	No report	Pictures of faces (visual)	Own infant > Unknown infant	46
Atzil [35]	2011	Maternal	23	4–6 months	22–37	Pictures of faces (visual)	Own infant > Unfamiliar infant	21
Barret [36]	2012	Maternal	22	3 months	25–35	Pictures of faces (visual)	Own infant > Unfamiliar infant	32
Wan [37]	2014	Maternal	20	6.2 months	32	Video Clip (visual-auditory)	Own infant > Unknown infant	40
**(B)**								
**First Author**	**Year**	**Categories of Love**	**Numbers of Participants (Female)**	**Mean Age of Participants**	**Experimental Stimuli**	**Contrasts**	**Numbers of Foci**	
Bartels & Zeki [21]	2000	Passionate	17 (11)	24.5	Pictures of faces (visual)	Lover > Familiar friend	13	
Aron [38]	2005	Passionate	17 (10)	20.6	Picture (visual)	Lover > Familiar friend	8	
Ortigue [39]	2007	Passionate	36 (36)	20.1	Words (visual)	Lover > Familiar friend Lover > Noun	14 4	
Kim [40]	2009	Passionate	10 (5)	21.1	Pictures of faces (visual)	Lover > Familiar friend	29	
Zeki [41]	2010	Passionate (opposite and same sex)	24 (12)	26.3	Pictures of faces (visual)	Lover > Familiar friend	11	
Stoessel [42]	2011	Passionate	12 (6)	24.08	Picture (visual)	Lover > Erotic pictures	16	
Xu [43]	2011	Passionate (Chinese participants)	18 (10)	21.61	Pictures of faces (visual)	Lover > Familiar friend	8	
Xu [44]	2012	Passionate	18 (0)	25.11	Picture (visual)	Lover > Familiar friend	10	
Acevedo [26]	2012	Passionate (long-term)	17 (10)	52.85	Pictures of faces (visual)	Lover > Familiar acquaintance Lover > Close friend	30 26	

**Table 2:** Coordinates of the maxima in the activated brain regions associated with maternal love.

Brain Regions	BA	MNI Coordinates			Voxels	Extrema Value
		X	Y	Z		
Left ventral tegmental area	-	−2 0	−14 −24	−20 −12	360 488	0.02679 # 0.03247 *
Right thalamus	-	10 4	−18 −8	0 −2	440 80	0.04450 * 0.03028 *
Left substantia nigra	-	−8	−24	−14	264	0.03697 *
Left putamen	-	−22 −28	4 −14	0 −4	240 184	0.04104 * 0.03775 *
Right medial prefrontal gyrus	10	8	64	14	144	0.03046 #
Right superior temporal gyrus	21	58	−6	−18	80	0.02490 #
Right middle temporal gyrus	21	62	−4	−14	312	0.02606 #
Right putamen	-	28	−16	6	120	0.03611 #
Left amygdala	-	−32	−6	−18	312	0.03551 #

Threshold: FWE ≤ 0.05 *, FDR pN ≤ 0.01 #, volume > 200 mm^3^. BA, Brodmann area.

**Table 3:** Coordinates of the maxima in the activated brain regions associated with passionate love.

Brain Regions	MNI Coordinates			Voxels	Extrema Value
	X	Y	Z		
Left ventral tegmental area	−2	−14	−10	184	0.03104 *
Right ventral tegmental area	2	−22	−16	480	−0.02994 *

Threshold: FWE *p* ≤ 0.05 *, volume > 200 mm^3^.

**Table 4:** Coordinates of the maxima in the activated brain regions, contrasting between maternal and passionate love across studies.

Brain Regions	MNI Coordinates			Voxels	Extrema Value
	X	Y	Z		
*Maternal > Passionate love*					
Left putamen	−22	4	−4	184	0.02739
*Passionate > Maternal love*					
No significance	-	-	-	-	-

Threshold: FDR pN ≤ 0.01, volume > 200 mm^3^. BA, Brodmann area.

## Data Availability

Not applicable.
